# Editorial: Metabolic engineering of valuable compounds in photosynthetic organisms

**DOI:** 10.3389/fpls.2023.1260454

**Published:** 2023-09-05

**Authors:** Zhi-Yan Du, Wajid Waheed Bhat, Guoyin Kai, Inna Khozin-Goldberg, Xiao-Hong Yu, Agnieszka Zienkiewicz, Krzysztof Zienkiewicz

**Affiliations:** ^1^ Department of Molecular Biosciences and Bioengineering, University of Hawaii at Manoa, Honolulu, HI, United States; ^2^ Plant Biotechnology and Agrotechnology Division, Council of Scientific and Industrial Research-Indian Institute of Integrative Medicine (CSIR-IIIM), Jammu, India; ^3^ Laboratory of Medicinal Plant Biotechnology, College of Pharmacy, Zhejiang Chinese Medical University, Hangzhou, Zhejiang, China; ^4^ French Associates Institute for Agriculture and Biotechnology of Drylands, Jacob Blaustein Institutes for Desert Research, Ben-Gurion University of the Negev, Sede Boqer, Israel; ^5^ Biology Department, Brookhaven National Laboratory, Upton, NY, United States; ^6^ Centre for Modern Interdisciplinary Technologies, Nicolaus Copernicus University in Toruń, Toruń, Poland

**Keywords:** metabolic engineering, synthetic biology, plants and algae, natural products, secondary metabolites, genetic modification, breeding

Photosynthetic organisms, including plants and algae, possess a remarkable ability to harness carbon dioxide and solar energy, enabling them to produce a vast array of complex compounds such as phenolic acids (Zhou et al., 2021), terpenes (Miller et al., 2020), unsaturated fatty acids (Kokabi et al., 2020; Gan et al., 2022), and other lipid products (Zienkiewicz and Zienkiewicz, 2020). This inherent capability positions them as highly promising platforms for the sustainable production of valuable biomolecules. While the industrial application of photosynthetic organisms in synthetic biology is not as advanced as that of model heterotrophs or mammalian systems, their significance as primary contributors to global biomass can be further developed. In fact, they are increasingly emerging as key players in the booming field of synthetic bioproducts, driven by advancements in genome editing tools and other innovative technologies. As we explore and exploit the potential of photosynthetic organisms, we open up exciting possibilities for the production of environmentally friendly and renewable biomaterials that can address pressing societal and ecological challenges.

This Research Topic includes eight original research and two review articles, with a special focus on the metabolic engineering of valuable biomaterials in plants and algae. Taparia et al., developed modular CRISPR/Cas9 constructs for the model diatom *Phaeodactylum tricornutum* that allow the multiplexed targeting and creation of marker-free genome-edited lines. The system was used to knock out *StLDP*, the gene encoding Stramenopile-type lipid droplet protein essential for lipid droplet biogenesis (**Figure 1**). Mellor et al. expressed human P450s in tobacco chloroplasts to produce indican, suggesting a strategy for producing high-value chemicals or drug metabolites in photosynthetic organisms (**Figure 1**). Another research ariticle investigated the biosynthesis of isoprenoids in poplar, and revealed that the 3-hydroxy-3-methylglutaryl-CoA reductase (HMGR) and 1-deoxy-D-xylulose5-phosphate reductoisomerase (DXR) play important roles in regulating the genes in methylerythritol phosphate (MEP) and mevalonic acid (MVA) pathways and isoprenoids made from the MEP and MVA pathways (**Figure 1**) (Movahedi et al.). Li et al. produced carotenoids in rice (*Oryza sativa*) endosperm by overexpressing rice GOLDEN2-LIKE (*OsGLK*) transcription factor and *OsGLK* with three other carotenogenic genes, *tHMG1* (truncated *Saccharomyces cerevisiae* 3-hydroxy-3-methylglutaryl-CoA reductase), *ZmPSY1* (*Zea mays* L. phytoene synthase), and *PaCrtI* (*Pantoea ananatis* phytoene desaturase), to improve the nutritional composition of rice (**Figure 1**). Another research article in rice developed models by multilevel mathematical modeling using the data from rice lines with genome modification in MVA pathways, providing tools that can help prioritize metabolic engineering strategies for specific metabolic goals through exogenous pathways (**Figure 1**) (Basallo et al.). In perennial herbs, Wang et al. identified physiological/biochemical indicators, such as enzyme activities of glutamine synthetase (GS), glutamate synthase (GLS), glutamate dehydrogenase (GDH), peroxidase (POD), and catalase (CAT), were related to biomass accumulation in *Salvia miltiorrhiza* (**Figure 1**); Su et al. characterized the *β*-glucosidase in *Platycodon grandifloras*, which can convert glycosylated platycoside E to Platycodin D *in vitro* (**Figure 1**); Jin et al. identified an MYB transcription factor OvMYBPA2 in *Onobrychis viciifolia* by transcriptome analyses and confirmed its function in the regulation of proanthocyanidins in transgenic *Medicago sativa* (**Figure 1**). Strand and Walker reviewed bioengineering from an energetics perspective using photosynthetic organisms for bioproducts of interest (**Figure 1**). Another review article discussed the recent progress in engineering fatty acids and storage lipids in various plant species and tissues and summarized an inventory of specific lipogenic factors for plant lipid products (**Figure 1**) (Cai et al.).

**Figure 1:**
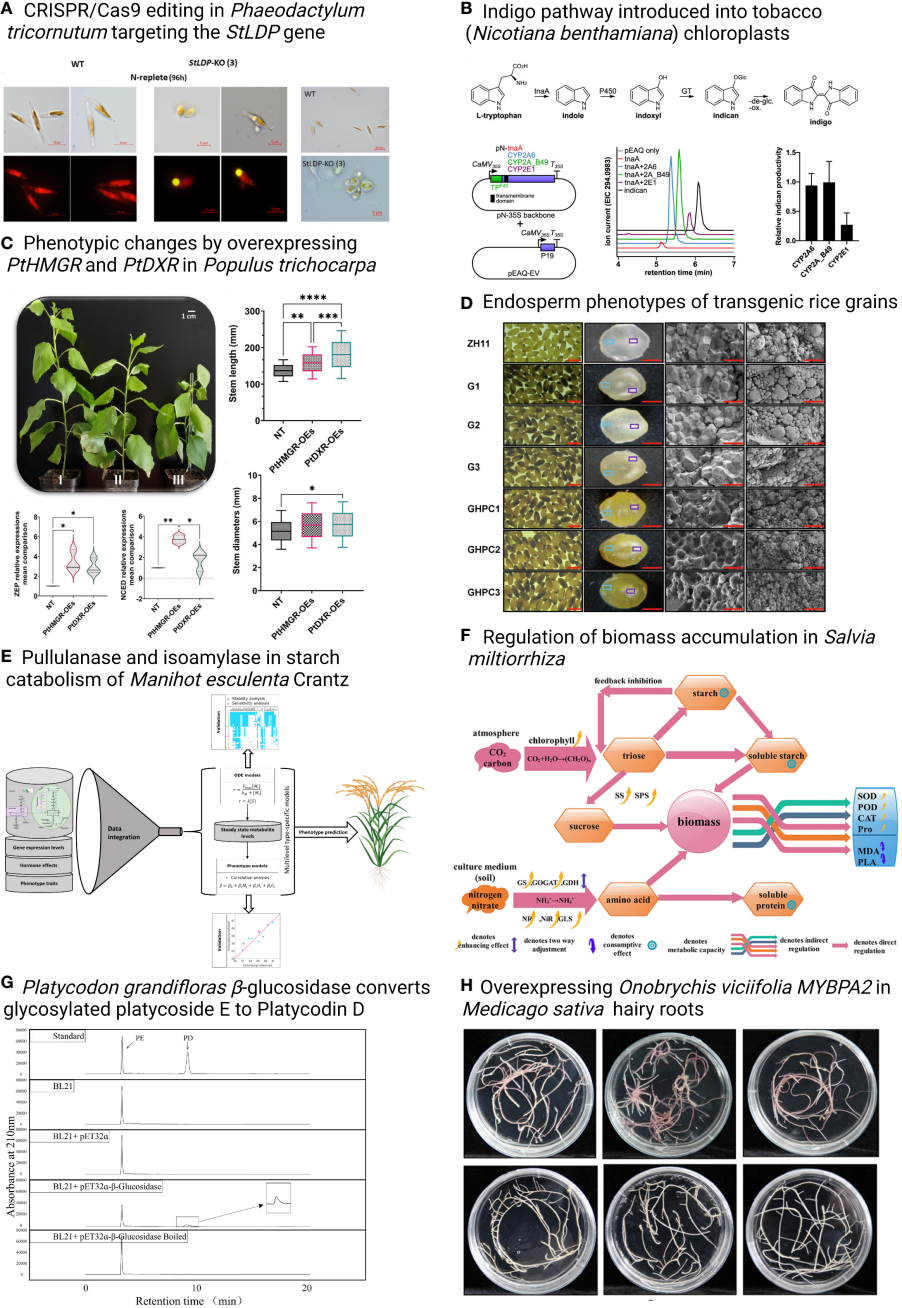
Overview of the original research articles in this Synthetic Biology Research Topic. Asterisks indicate statistically significant differences, *p <0.05, **p <0.01, ***p <0.001, ****p <0.0001

## Author contributions

Z-YD: Funding acquisition, Resources, Visualization, Writing – original draft, Writing – review & editing. WB: Writing – review & editing. GK: Writing – review & editing. IK-G: Writing – review & editing. X-HY: Writing – review & editing. AZ: Writing – review & editing. KZ: Writing – review & editing.
